# Repeatability of corneal measurements from the Casia2 anterior segment tomographer in a cataractous population

**DOI:** 10.1371/journal.pone.0328894

**Published:** 2025-07-23

**Authors:** Achim Langenbucher, Nóra Szentmáry, Alan Cayless, Daniel Schartmüller, Marcus Lisy, Rupert Menapace, Jascha Wendelstein, Christina Leydolt

**Affiliations:** 1 Department of Experimental Ophthalmology, Saarland University, Homburg/Saar, Germany; 2 Dr. Rolf M. Schwiete Center for Limbal Stem Cell and Aniridia Research, Saarland University, Homburg/Saar, Germany; 3 Department of Ophthalmology, Semmelweis-University, Budapest, Hungary; 4 School of Physical Sciences, The Open University, Milton Keynes, United Kingdom; 5 Department of Ophthalmology and Optometry, Vienna University Hospital, Vienna, Austria; 6 Department of Ophthalmology, Ludwig Maximilian University (LMU), Munich, Germany; Instituto Superior de educação e Ciencias, ISEC Lisboas, PORTUGAL

## Abstract

**Purpose:**

The purpose of this study was to investigate the repeatability of corneal power and thickness measures in a large patient cohort.

**Methods:**

In this retrospective non-randomised cross sectional single-centre study we evaluated a dataset containing 900 Casia2 anterior segment tomography measurements from 300 eyes from 300 patients (3 repeat measurements each) taken prior to cataract surgery. Only complete measurements marked as ‘Successful’ and with a sequence of 3 measurements for each eye performed on the same day were considered. Keratometry (K), corneal back surface (P) and RealPower (R) were each decomposed into power vector components SEQ/C0/C45. The mean (MEAN) and standard deviations (SD) for each sequence of measurements were derived and analysed.

**Results:**

For K the MEAN and SD SEQ/C0/C45 were 43.77/0.17/-0.02 D and 0.12/0.18/0.18 D, for P MEAN and SD were −6.13/-0.25/0.00 D and 0.01/0.03/0.02 D, and for R MEAN and SD were 42.77/-0.06/-0.03 D and 0.13/0.19/0.19 D. For K and R 20%/42%/41% and 24%/45%/44% of repeat measurements respectively fell outside limits of ±1/8 D. The cornea was slightly thicker at the apex than at the thinnest point (544.6 vs. 536.2 µm) and SD was 4.3 vs. 3.9 µm. Keratometry underestimates against-the-rule corneal astigmatism by around 0.23 D.

**Conclusions:**

The variation of corneal power with repeat measurements is in a range of ±1/8 D for keratometric and RealPower SEQ. Where the Casia2 is used for lens power calculation, repeat measurements could help to improve the refractive outcome.

## Introduction

In cataract surgery, intraocular lens (IOL) power calculation requires reliable measurement data on corneal geometry [1,2]. However, corneal power cannot be measured directly, therefore we have to determine corneal power from the shape of the corneal front surface using (automated) keratometry or corneal topography, or from the shape of both corneal surfaces together with corneal thickness using tomography [3–5]. In classical keratometry or topography, a keratometer index is used for conversion of corneal front surface radius into corneal power [5]. However, we know that such a keratometer index is based on several model assumptions such as a fixed front to back surface curvature ratio and other simplifications which may not be consistently applicable based on corneal front surface measurement only [6]. In addition to variation of front to back surface curvature ratio, we know from Javal’s rule [7–9] that the corneal back surface curvature does not show fixed proportionality to corneal front surface curvature in all meridians, with the consequence that corneal back surface adds some astigmatism against the rule which is not explained by keratometry or corneal topography. Where the power of toric lens implants is determined based on keratometry, we typically use nomogram or regression corrections to consider the portion of corneal back surface astigmatism which is not described by keratometry [3,4].

In addition to Scheimpflug tomography, several anterior segment optical coherence tomographers (OCT) have been established on the market. These promise excellent axial and lateral resolution [10–25]. From the literature we know that reliable corneal measures are mandatory for lens power calculation, especially in short eyes, and that keratometry seems to be the most critical measure preventing us from reaching our goal of target refraction [1,2] in the current state of the art in optical biometry.

Manual keratometry evaluates the corneal shape at 2 distinct locations in the mid periphery of the cornea, in both the flat and steep meridians. Automated keratometry as integrated in automatic refracto-keratometers or biometers typically evaluates corneal shape in more than 2 meridians or multiple zones, whereas simulated keratometry values derived from Placido topographers aim to mimic manual keratometry by analysing a specific ring for the flat and steep meridians together with the orientation axis [18,26]. In contrast, there are techniques which evaluate corneal curvature in larger areas to obtain more robust data even with corneal irregularities or scars. Techniques such as Fourier decomposition of ring-shaped corneal power data or Zernike decomposition of corneal height data may overcome the limitations of deriving data from distinct locations and thus provide more robust information on corneal curvature [5]. Where measurements from both corneal surfaces are available, this eliminates the need for the use of a keratometer index along with its limitations. In addition to corneal front surface curvature or (keratometric or front surface) power, information is also provided on the corneal back surface curvature or power, with total power values (e.g., RealPower) typically being calculated from corneal front and back surface curvature using the thick lens formula (based on linear Gaussian optics).

In the standard notation, corneal power is represented by its spherical power, astigmatism and the orientation axis of astigmatism. However, as the astigmatism axis shows periodicity with 180 degrees and the reliability of the axis drops systematically for small values of astigmatism [3,4], power vector components in terms of the spherical equivalent power and the projections of astigmatism to the 0/180 degree and 45/135 degree axis (Humphrey notation) seem to be more appropriate to describe corneal shape.

### The purpose of the present study was

- to perform a sequence of 3 repeat measurements with the Casia2 anterior segment tomographer in a cataractous population,
- to decompose simulated keratometry derived from corneal front surface (Ka), corneal back surface curvature (Kp) and RealPower values (Kr), and the respective data from Fourier decomposition within the central 3 mm zone (Kf3a, Kf3p, Kf3r) and 6 mm zone (Kf6a, Kf6p, Kf6r) of the cornea into power vector components,
- to calculate the mean values of the repeat measurements and the deviations of the repeat measurements from the corresponding mean value, and to extract statistically relevant repeatability metrics such as the within subject standard deviation (Sw).

## Methods

### Dataset for our evaluation

A dataset containing 3 repeat measurements for each of 308 eyes (in total N = 924 measurements) taken prior to cataract surgery scheduled for implantation of a non-toric intraocular lens and without a history of previous eye surgery was considered in this study. All measurements were performed between February 23 2023 and December 06 2023 at the Department of Ophthalmology and Optometry, Medical University Vienna (Vienna, Austria) with the Casia2 anterior segment optical coherence tomography device (Tomey, Nagoya, Japan).

The data were anonymised at source and transferred to a.csv data table using the software module for batch data export. The local ethics committee (IRB) has provided a waiver for this study (Ärztekammer des Saarlandes, 157/21), as all data processed in this study were already anonymised at source before being transferred to us for processing. This precludes any back-tracing of the identity, and therefore informed consent of the patients was not necessary. Data tables were reduced to the relevant parameters required for our data analysis, consisting of the following measurements: patient ID, exam date and time, the laterality (left or right eye), (keratometric) corneal front surface power in the flat (K1a) and steep (K2a) meridians, both in D at axis A1a and A2a in degrees, corneal back surface power in the flat (K1p) and steep (K2p) meridians, both in D at axis A1p and A2p in degrees, RealPower in the flat (K1r) and steep (K2r) meridians, considering both corneal surfaces in terms of a thick lens model of the cornea both in D at axis A1r and A2r in degrees. These measurements are understood to be derived from the 3 mm zone. Additionally, the dataset also contains the corresponding keratometric (K1f3a, K2f3a, A1f3a, A2f3a), back surface (K1f3p, K2f3p, A1f3p, A2f3p) and RealPower (K1f3r, K2f3r, A1f3r, A2f3r) data from Fourier analysis of corneal power data derived from the central 3 mm zone, and the respective keratometric (K1f6a, K2f6a, A1f6a, A2f6a), back surface (K1f6p, K2f6p, A1f6p, A2f6p) and RealPower (K1f6r, K2f6r, A1f6r, A2f6r) data from Fourier analysis of corneal power data derived from the central 6 mm zone, and corneal thickness at the apex (CCT) and at the thinnest point of the cornea (TCT), both in microns.

From the dataset we considered eyes where the 3 repeat measurements were taken within a time period of 1 hour. Subjects with missing data or data with a ‘Failed’ or ‘Warning’ in the internal quality check in the CCT or TCT or in the keratometric, back surface, or RealPower in one or more measurements were omitted from the dataset. The data were transferred to Matlab (Matlab 2022b, MathWorks, Natick, USA) for further processing.

### Data pre-processing in Matlab

Corneal front surface data, back surface data, and RealPower data were converted into power vector components with spherical equivalent power derived from the arithmetic mean of K1 and K2 (SEQ: = 0.5·(K1 + K2) and the projections of the astigmatism (K2-K1) to the 0°/90° meridian (C0:= (K2-K1)·cos(2·A1)) and to the 45°/135° meridian (C45: = (K2-K1)·sin(2·A1)). At the end we considered the power vector component triplets for keratometry (KaSEQ/KaC0/KaC45, Kf3aSEQ/Kf3aC0/Kf3aC45, Kf6aSEQ/Kf6aC0/Kf6aC45), for corneal back surface power (KpSEQ/KpC0/KpC45, Kf3pSEQ/Kf3pC0/Kf3pC45, Kf6pSEQ/Kf6pC0/Kf6pC45), and for RealPower (KrSEQ/KrC0/KrC45, Kf3rSEQ/Kf3rC0/Kf3rC45, Kf6rSEQ/Kf6rC0/Kf6rC45).

In the next step we derived the mean values for all power vector components and for CCT and TCT for the sequence of 3 repeat measurements (indicated by (.)m) and the deviation of all repeat measurements from the mean of the 3 repeat measurements (indicated by (.)d).

### Data processing in Matlab and statistics

The descriptive statistics for the mean (.)m and deviation (.)d values are summarised in tables in terms of arithmetic mean, standard deviation, median, and the lower and upper boundaries of the 95% confidence interval (2.5% and 97.5% quantile). The within-subject standard deviation Sw were derived from the variations of the sequence of 3 repeat measurements per eye. The distributions for the CCTm/CCTd and TCTm/TCTd values are shown as boxplots. For the power vector components we used boxplots to display the distribution of the SEQ components and double angle plots with the C45 component in Y direction and the C0 component in X direction to show the scatter of the astigmatism [3–5]. Since we expect mirror symmetry in the astigmatism for left and right eyes with respect to the vertical axis (indicated by a C45 component flipped in sign for left and right eyes), we plotted the scatter for right and left eyes separately.

The Shapiro-Wilk test or the Henze-Zirkler test was used to test for univariate (CCT, TCT and SEQ) or multivariate (SEQ and astigmatic power vectors with components C0 and C45) parameters [27,28]. In the case of univariate normality we used the t-test for paired samples, and in the case of multivariate normality we calculated the centroid and 95% error ellipses based on the variance-covariance matrix, and the parametric Hotelling T2 test was used to test for paired differences. In the case of univariate non-normality we used the Wilcoxon test for paired samples, and in the case of multivariate non-normality we calculated the medoid and 95% confidence region based on iterative convex hull stripping techniques, and the nonparametric multivariate rank sign test was used to test for paired differences [29–32].

## Results

From the N = 924 Casia2 measurements transferred to us, and after considering the selection criteria, a total of N = 900 measurements (N = 300 eyes) were selected for our analysis (152 right and 148 left eyes from 168 female and 132 male patients).

Table 1 shows the mean values of the 3 repeat measurements for the power vector components for keratometry, corneal back surface and RealPower, together with the corresponding power vector components derived from Fourier analysis in the central 3 mm and 6 mm zone. In Table 2 the explorative data of the deviations of the 3 repeat measurements from the mean values are listed for the power vector components for keratometry, corneal back surface and RealPower, together with the corresponding power vector components derived from Fourier analysis in the central 3 mm and 6 mm zone.

**Table 1:** Explorative data for the mean values of corneal power derived from 3 repeat measurements prior to cataract surgery, including keratometry, corneal back surface power, and RealPower decomposed into power vector components (upper block) in terms of spherical equivalent power (SEQ), and the projections of the astigmatism to the 0°/90° axis (C0) and to the 45°/135° axis (C45). In the middle and lower block the corresponding data are shown for the Fourier analysis within the central 3 mm and 6 mm zones. SD refers to the standard deviation, and the 2.5% quantile and 97.5% quantile to the lower and upper boundaries of the 95% confidence intervals.

Mean from 3 repeat measurements (N = 300 eyes)		Keratometry (corneal front surface)			Corneal back surface			RealPower considering front and back surface		
Data in D		KaSEQ	KaC0	KaC45	KpSEQ	KpC0	KpC45	KrSEQ	KrC0	KrC45
Keratometry, corneal back surface, RealPower	Mean	43.7761	0.1530	−0.0384	−6.1255	−0.2499	0.0042	42.7684	−0.0712	−0.0387
	SD	1.5395	0.5908	0.3716	0.2390	0.1201	0.0957	1.5158	0.5939	0.3892
	Median	32.8473	0.1419	−0.0492	−6.1210	−0.2525	0.0040	42.8475	−0.0946	−0.0621
	2.5% quantile	40.0519	−1.0162	−0.8019	−6.6106	−0.4785	−0.1467	39.0200	−1.1986	−0.8181
	97.5% quantile	46.7690	1.2772	0.7676	−5.6113	−0.0205	0.1829	45.7150	1.0532	0.7416
Data in D		Kf3aSEQ	Kf3aC0	Kf3aC45	Kf3pSEQ	Kf3pC0	Kf3pC45	Kf3rSEQ	Kf3rC0	Kf3rC45
Fourier analysis in a central 3 mm zone	Mean	43.9684	0.2281	0.0142	−6.1174	−0.2542	−0.0006	42.9858	0.0007	0.0156
	SD	1.5605	0.6250	0.4382	0.2381	0.1200	0.0974	1.5423	0.6341	0.4612
	Median	44.0900	0.2542	−0.0063	−6.1183	−0.2507	−0.0031	43.1117	−0.0171	−0..0088
	2.5% quantile	40.2933	−1.0488	−0.7178	−6.6067	−0.4770	−0.1556	39.4067	−1.3212	−0.8094
	97.5% quantile	46.8633	1.3808	0.9838	−5.5900	−0.0222	0.1914	45.6600	1.2016	1.0274
Data in D		Kf6aSEQ	Kf6aC0	Kf6aC45	Kf6pSEQ	Kf6pC0	Kf6pC45	Kf6rSEQ	Kf6rC0	Kf6rC45
Fourier analysis in a central 6 mm zone	Mean	43.7211	0.1484	−0.0187	−6.0891	−0.2248	0.0021	42.7403	−0.0629	−0.0161
	SD	1.5302	0.6269	0.4037	0.2344	0.1073	0.0970	1.5052	0.6307	0.4208
	Median	43.7767	0.1790	−0.0445	−6.0883	−0.2311	0.0035	42.8133	−0.0908	−0.0526
	2.5% quantile	39.9033	−1.0862	−0.7316	−6.5667	−0.4239	−0.1740	39.1067	−1.2620	−0.7828
	97.5% quantile	46.6800	1.3436	0.8466	−5.5600	0.0073	0.1772	45.6300	1.1233	0.8906

**Table 2:** Explorative data for the deviation of the 3 individual repeat measurements from the mean value of the 3 repeat measurements, including keratometry, corneal back surface power, and RealPower decomposed into power vector components (upper block) in terms of spherical equivalent power (SEQ), and the projections of the astigmatism to the 0°/90° axis (C0) and to the 45°/135° axis (C45). In the middle and lower block the corresponding data are shown for the Fourier analysis within the central 3 mm and 6 mm zones. The mean deviation is not shown in the table as it equals zero for all parameters. SD refers to the standard deviation, and the 2.5% quantile and 97.5% quantile to the lower and upper boundaries of the 95% confidence intervals. Table cells in bold letters indicate the within-subject standard deviations Sw. The portion of repeat measurements outside the clinically relevant threshold of ±1/8 D from the mean value is shown in the last row of each block.

Deviation of the 3 repeat measurements from the mean value (N = 900 measurements)		Keratometry (corneal front surface)			Corneal back surface			RealPower considering front and back surface		
Data in D		KaSEQ	KaC0	KaC45	KpSEQ	KpC0	KpC45	KrSEQ	KrC0	KrC45
Keratometry, corneal back surface, RealPower	SD	**0.0891**	**0.1443**	**0.1412**	**0.0063**	**0.0163**	**0.0167**	**0.0968**	**0.1512**	**0.1480**
	Median	0.0009	−0.0007	−0.0037	0.0000	−0.0001	0.0000	0.0017	0.0000	−0.0002
	2.5% quantile	−0.1562	−0.2781	−0.2995	−0.0133	−0.0300	−0.0334	−0.1700	−0.2859	−0.3025
	97.5% quantile	0.1583	0.2766	0.2908	0.0133	0.0316	0.0346	0.1717	0.2942	0.3046
	% ≥ ±1/8 D	10.11	33.67	34.11	0.00	0.11	0.00	12.00	36.44	35.11
Data in D		Kf3aSEQ	Kf3aC0	Kf3aC45	Kf3pSEQ	Kf3pC0	Kf3pC45	Kf3rSEQ	Kf3rC0	Kf3rC45
Fourier analysis in a central 3 mm zone	SD	**0.1543**	**0.1897**	**0.1786**	**0.0069**	**0.0158**	**0.0168**	**0.1688**	**0.2080**	**0.1982**
	Median	0.0067	−0.0042	0.0033	0.0000	−0.0001	0.0002	0.0067	0.0040	0.002
	2.5% quantile	−0.3300	−0.3712	−0.3525	−0.0133	−0.0318	−0.0330	−0.3633	−0.4048	−0.3726
	97.5% quantile	0.3100	0.3765	0.3458	0.0133	0.0307	0.0338	0.3400	0.4172	0.3938
	% ≥ ±1/8 D	30.33	45.56	44.44	0.00	0.11	0.00	34.89	50.89	49.11
Data in D		Kf6aSEQ	Kf6aC0	Kf6aC45	Kf6pSEQ	Kf6pC0	Kf6pC45	Kf6rSEQ	Kf6rC0	Kf6rC45
Fourier analysis in a central 6 mm zone	SD	**0.0898**	**0.1462**	**0.1532**	**0.0061**	**0.0178**	**0.0161**	**0.0976**	**0.1557**	**0.1677**
	Median	0.0000	0.0018	−0.0005	0.0000	−0.0002	0.0005	0.0033	0.0017	0.0026
	2.5% quantile	−0.1700	−0.3108	−0.3017	−0.0100	−0.0275	−0.0330	−0.1900	−0.2919	−0.3298
	97.5% quantile	0.1533	0.2948	0.3154	0.0100	0.0305	0.0332	0.1667	0.3461	0.3573
	% ≥ ±1/8 D	10.67	31.78	35.00	0.00	0.22	0.00	12.56	34.89	37.89

Table 3 lists the descriptive data of CCT and TCT in terms of mean, standard deviation, median and the 95% confidence intervals with the mean values (.)m of the 3 repeat measurements listed in the left two columns and the deviations of the 3 repeat measurements from the mean value (.)d interval in the two columns on the right. As shown in Fig 3, the mean corneal thickness at the thinnest point is statistically significantly lower than the mean corneal thickness at the apex. The variation of the 3 repeat measurements seems to be slightly higher for corneal thickness at the thinnest point compared to the variation of the 3 repeat measurements at the corneal apex.

**Table 3:** Explorative data of corneal thickness at the apex (CCT) and at the thinnest point of the cornea (TCT) in terms of mean, standard deviation, median and the lower (2.5% quantile) and upper boundary (97.5% quantile) of the 95% confidence interval. In the left block we displayed the mean values for the 3 repeat measurements (indicated by (.)m), and on the right block the deviations of the 3 repeat measurements from the mean value (indicated by (.)d). Table cells in bold letters indicate the within-subject standard deviations Sw. The portion of repeat measurements outside the clinically relevant threshold of ±5 micron from the mean value is shown in the last row.

Corneal thickness in microns	Mean of the 3 repeat measurements (N = 300 eyes)		Deviation of the 3 repeat measurements from the mean value (N = 900 measurements)	
	CCTm	TCTm	CCTd	TCTd
Mean	544.1	535.7	0	0
Standard deviation	34.9	36.4	**1.0**	**1.1**
Median	542.0	534.3	0.0	0.0
2.5% quantile	482.7	464.3	−1.7	−2.0
97.5% quantile	624.0	616.0	1.7	2.0
% ≥ ±5 microns			0.2	0.2

**Fig 1:**
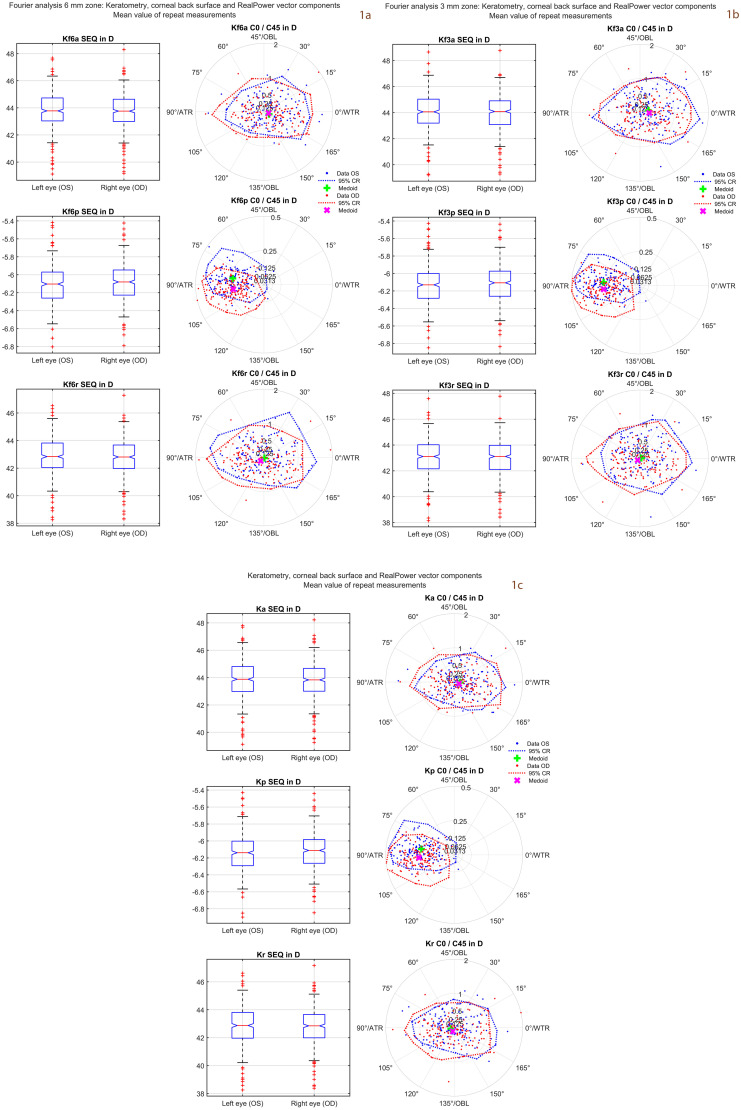
Distributions of the mean values from the 3 repeat measurements for the corneal power vector components (N = 300 eyes). In the 3 subfigures the upper graphs refer to keratometry (corneal front surface power), the middle graphs to corneal back surface power, and the lower graphs to RealPower. On the left side of each graph the boxplots are displayed for the spherical equivalent power SEQ, and on the right side the scatter of the 2 astigmatic power vector components C0 and C45 is shown using double angle plots. Subfigure 1a represents the data for keratometry, corneal back surface and RealPower, subfigure 1b represents the corresponding data derived from Fourier analysis in a central 3 mm zone of the cornea, and subfigure 1c the corresponding data derived from Fourier analysis in a central 6 mm zone of the cornea. Since none of the multivariate distributions showed normality the medoids and 95% confidence regions (CR) are displayed (instead of centroids and 95% error ellipses) based on iterative convex hull stripping techniques. Data are shown separately for left and right eyes, and mirror symmetry of corneal astigmatism with respect to the vertical axis refers to symmetry of the astigmatic power vector components with respect to the horizontal axis (C45 power vector components are flipped in sign for left and right eyes).

**Fig 2:**
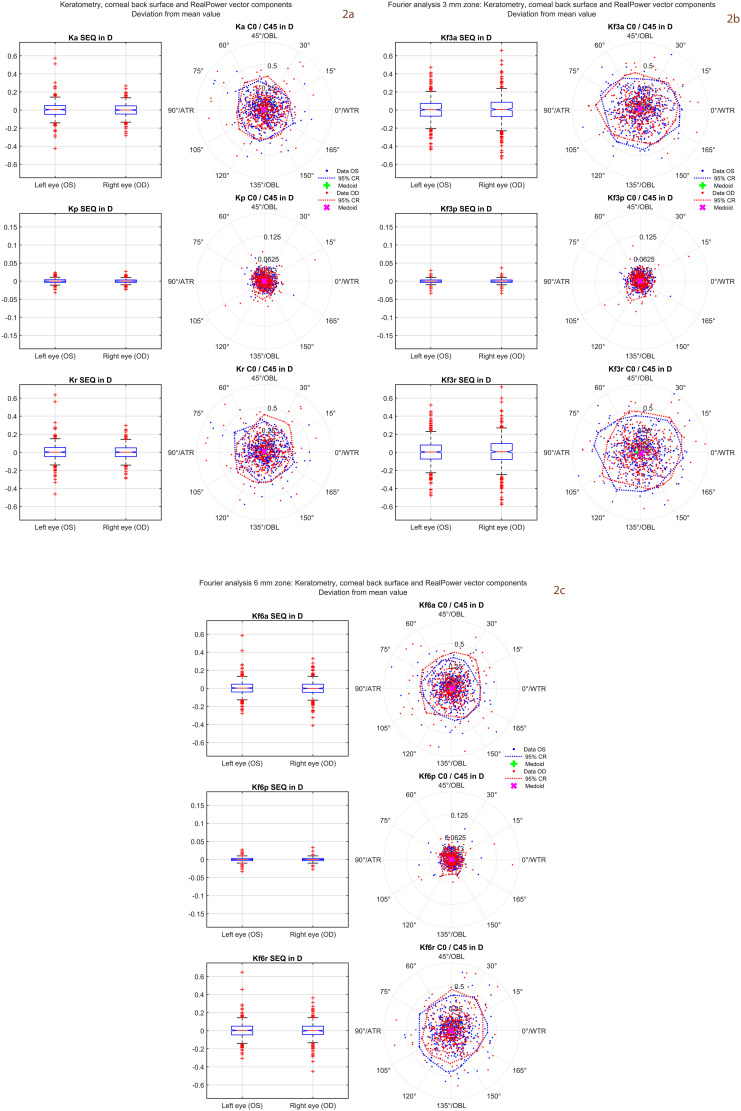
Distributions of the deviations of the 3 repeat measurements from the mean value of the 3 repeat measurements for the corneal power vector components (N = 900 measurements). In the 3 subfigures the upper graphs refer to keratometry (corneal front surface power), the middle graphs to corneal back surface power, and the lower graphs to RealPower. On the left side of each graph the boxplots are displayed for the spherical equivalent power SEQ, and on the right side the scatter of the 2 astigmatic power vector components C0 and C45 is shown using double angle plots. Subfigure 2a represents the data for keratometry, corneal back surface and RealPower, subfigure 2b represents the corresponding data derived from Fourier analysis in a central 3 mm zone of the cornea, and subfigure 2c the corresponding data derived from Fourier analysis in a central 6 mm zone of the cornea. Since none of the multivariate distributions showed normality the medoids and 95% confidence regions (CR) are displayed (instead of centroids and 95% error ellipses) based on iterative convex hull stripping techniques.

**Fig 3:**
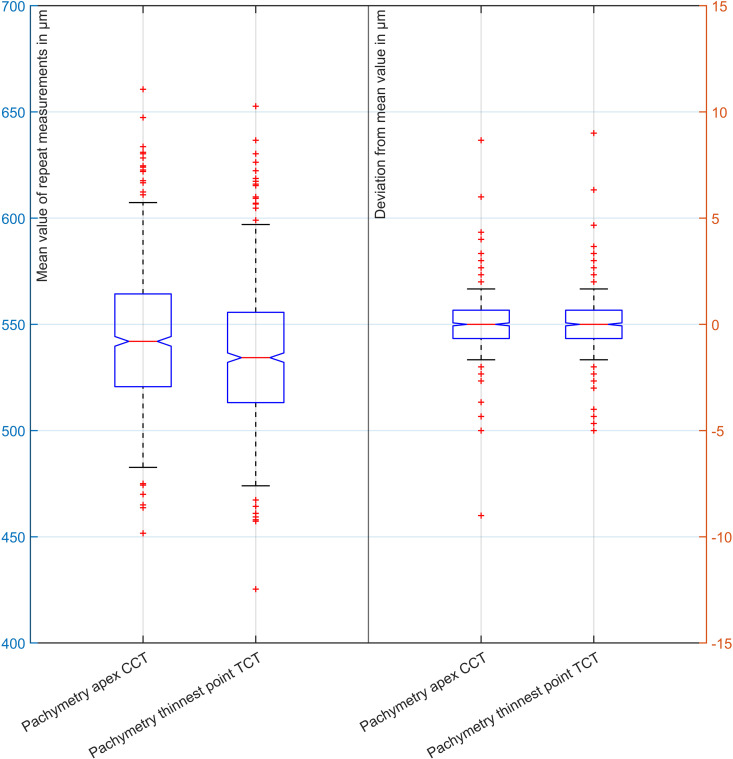
Boxplots showing the distributions of the mean values from the 3 repeat measurements of corneal thickness at the apex and at the thinnest point of the cornea (left side, y scale to the left) and the distributions of the deviations of the 3 repeat measurements from the mean value for the corneal thickness at the apex and at the thinnest point of the cornea (right side, y scale to the right). The mean corneal thickness at the thinnest point is statistically significantly lower than the mean corneal thickness at the apex. The variation of the 3 repeat measurements seems to be slightly higher for corneal thickness at the thinnest point compared to the variation of the 3 repeat measurements at the corneal apex.

Fig 1 displays the distributions of the mean values from the 3 repeat measurements of the power vector components for keratometry, corneal back surface power and RealPower (**subfigure 1a**) and the corresponding distributions of the power vector components derived from the Fourier analysis in a central corneal 3 mm zone (**subfigure 1b**) and in a central corneal 6 mm zone (**subfigure 1c**). The spherical equivalent power SEQ is shown using boxplots and the astigmatic power vector components are displayed in a polar double angle scatterplot with the C0 on the X axis and C45 on the Y axis. Data are separated for left and right eyes to give an impression of the symmetry conditions between left and right eyes. Since none of the multivariate distributions of the power vectors show normality the astigmatic power vectors are expressed in terms of medoids and 95% confidence regions.

Fig 2 displays the distributions of the deviations of the 3 repeat measurements from the mean values for the power vector components of keratometry, corneal back surface power and RealPower (**subfigure 2a**) and the corresponding distributions of the power vector components derived from the Fourier analysis in a central corneal 3 mm zone (**subfigure 2b**) and in a central corneal 6 mm zone (**subfigure 2c**). The spherical equivalent power SEQ is shown using boxplots and the astigmatic power vector components are shown in a polar double angle scatterplot with the C0 on the X axis and C45 on the Y axis. Data are separated for left and right eyes to give an impression of the symmetry conditions between left and right eyes. Since none of the multivariate distributions of the power vectors follow normality the astigmatic power vectors are expressed in terms of medoids and 95% confidence regions.

Fig 3 displays in the form of boxplots the distributions of the mean values from the 3 repeat measurements of corneal thickness at the apex and at the thinnest point of the cornea (left side, y scale to the left) together with the distributions of the deviations of the 3 repeat measurements from the mean value for the corneal thickness at the apex and at the thinnest point of the cornea (right side, y scale to the right). The mean corneal thickness at the thinnest point is statistically significantly thinner than the mean corneal thickness at the apex, but this difference of 8.4 microns on average does not seem to be clinically relevant.

## Discussion

Since the first launch of tomography for the anterior eye segment 20 years ago these instruments are increasingly involved in diagnosis, disease screening and monitoring, as well as in surgery planning. They are known to measure the corneal front and back surface and the corneal thickness profile with high precision [6,10–23,25,26]. However, there is no consensus as to which data provided by a tomographer are most representative for surgical planning, e.g., calculation of intraocular lens power. In addition to simulated keratometry values, which mostly mimic the measurement procedure with a manual keratometry, modern tomographers such as the Casia2 device provide curvature or power data for the corneal back surface, composite values representing the power of the cornea considered as a ‘thick lens’, and values derived from a decomposition of corneal power data into a Fourier series [33] or corneal height data into Zernike polynomials [34]. These decompositions include data from larger areas instead of local curvature or power values, promising more robust values compared to simulated keratometry data [34]. Corneal front surface data such as simulated keratometry values might not fully represent corneal power since variations in the anterior to posterior curvature ratio and corneal thickness are ignored.

In the present study we used a large dataset of Casia2 measurements taken prior to cataract surgery in a study population scheduled for implantation of a non-toric intraocular lens. This means that all of the eyes show low to moderate values of corneal astigmatism. For all eyes, a sequence of 3 repeat measurements was taken and before each measurement the patient’s head and the Casia2 device was re-adjusted. We analysed simulated keratometry values derived from the corneal front surface measurement (using a preset keratometry index of 1.3375), corneal back surface data, and RealPower values as composite data calculated from the corneal front and back surface measurements. In addition to the standard keratometry, back surface and RealPower data, the Casia2 software provides additional parameters which are derived from areal data in the central 3 mm zone and 6 mm zone of the cornea using a decomposition into a Fourier series [33]: The 0^th^ harmonic of the Fourier decomposition refers to the ‘mean power’ and the 2^nd^ harmonic to the regular portion of the corneal astigmatism within the region of interest. The 1^st^ harmonic of the Fourier decomposition refers to the asymmetry of corneal power, and the higher order harmonics (3^rd^ order and higher) represent mostly the local irregularity pattern of corneal power which might be of high interest for characterising local curvature fluctuations, e.g., in ectasia screening applications. We decided to use a representation of corneal power data in terms of the 3 power vector components (spherical equivalent power and the projections of the astigmatism to the 0°/90° and the 45°/135° meridian) instead of the standard notation as in our context the repeatability of the net corneal astigmatism as well as the axis orientation of corneal astigmatism seems to have no meaning. An analysis of the net corneal astigmatism may be problematic as this parameter ignores any rotation of the axis (e.g., if we compare an astigmatism of half dioptre oriented in 0° with an astigmatism of half dioptre oriented in 45°), and analysis of the axis orientation might ignore the 180-degree periodicity of the axis. In addition, we know from the literature that the axis variability increases systematically for low astigmatism (with a reciprocal dependency y ~ 1/x)), making definition of a consistent repeatability metric for the net astigmatism and the axis orientation difficult [4].

The data in Table 1 show that for our study population with low to moderate corneal astigmatism, the mean keratometric power (43.78 D) is quite similar to the mean keratometric powers derived with Fourier analysis in the 3 mm zone (43.96 D) and in the 6 mm zone (43.72 D). The mean corneal back surface power (−6.13 D) is quite similar to the mean corneal back surface powers derived with Fourier analysis in the 3 mm zone (−6.12 D) and in the 6 mm zone (−6.09 D).

The mean keratometric C0 vector component indicates an astigmatism with-the-rule of 0.15 D, and the corresponding values derived with Fourier analysis in the 3 mm zone (0.23 D) and in the 6 mm zone (0.15 D) are quite similar. The mean C0 vector component for the corneal back surface shows an astigmatism of −0.25 D against-the-rule (corresponding values derived with Fourier analysis in the 3 mm zone (−0.25 D) and in the 6 mm zone (−0.22 D)), which is not fully described with classical keratometry based on corneal front surface measurement.

The C0 vector component for RealPower (−0.07 D, corresponding values derived with Fourier analysis in the 3 mm zone (0.00 D) and in the 6 mm zone (−0.06 D)) differs considerably from the corresponding measures for keratometry. This means that a simplistic (constant) vector correction for the corneal back surface astigmatism should add 0.15 D – −0.07 D = 0.22 D against-the-rule astigmatism to keratometry (or add 0.23 D/ 0.21 D against-the-rule astigmatism to the keratometry values derived with Fourier analysis in the 3 mm/ 6 mm zone). In contrast, the mean C45 vector components for keratometry, corneal back surface power and RealPower (as well as the respective components from Fourier analysis in the 3 mm and 6 mm zone) are all close to zero (between −0.04 and 0.02 D).

The data listed in Table 2 give some insight into the variability of repeat measurements with the Casia2 anterior segment tomographer. The standard deviations (equivalent to within-subject standard deviations Sw) indicate that the power vector components (SEQ/C0/C45) variations in keratometry (0.09/0.14/0.14 D) are quite similar to the variations in RealPower (0.10/0.15/0.15 D) and much higher than variations in corneal back surface power (0.01/0.02/0.02 D). However, it was surprising to us that the corresponding variations in the keratometry and RealPower power vector components derived from Fourier analysis in the 3 mm zone (0.15/0.19/0.18 D and 0.17/0.21/0.20 D) are larger compared to the respective Sw values for standard keratometry of keratometry from the Fourier analysis in the 6 mm zone (0.09/0.15/0.15 D and 0.10/0.16/0.17 D), whereas variations in corneal back surface vector components or in all power vector components derived from Fourier analysis in the 6 mm zone are comparable. This means that, even if Fourier decomposition promises to better represent the ‘regular portion’ of corneal astigmatism (e.g., to be corrected with toric intraocular lenses or spherocylindrical glasses), it might be less repeatable at least in the 3 mm zone. Assuming that corneal power or astigmatism could be measured clinically with a resolution of 1/8 D = 0.125 D, we see that keratometric/ RealPower SEQ shows a larger variation in 10%/12% of repeat measurements whereas the corneal back surface SEQ is consistently within limits. Keratometric/ RealPower C0 and C45 show a larger variation in 34%/36% and 34%/35% of repeat measurements and the corneal back surface C0 and C45 values are again nearly consistently within limits (both less than 1%). For keratometry and RealPower derived from Fourier analysis in the 3 mm zone the portion of SEQ, C0 and C45 repeat measurements outside the limits is systematically larger, whereas for the Fourier analysis in the 6 mm zone the portion of repeat measurements outside limits is comparable to classical keratometry.

Fig 1 shows that there is no systematic difference in keratometric, corneal back surface, or RealPower SEQ between left and right eyes. From the locations of the medoids and confidence regions we see that, especially for the corneal back surface (middle right graphs in subfigures a, b and c), the left and right eyes are somewhat (but not perfectly) mirrored with respect to the X axis indicating mirror symmetry of corneal back surface astigmatism with respect to a vertical facial symmetry axis. This mirror symmetry seems to be much less pronounced for the keratometry (upper graphs) or RealPower (lower graphs). It might be important to note again that none of the power vectors follow multivariate normality, and therefore parametric statistics evaluating the centroid of the distributions or the error ellipses derived from variance-covariance matrix might be inappropriate. Instead, nonparametric statistics evaluating the multivariate medoids together with confidence regions (e.g., using iterative convex hull stripping) could be used instead [27,29–32].

Fig 2 shows the variations of the repeat measurements from the mean value. The distributions of the SEQ power vector components seem to be quite symmetric with respect to their mean or median value. However, for the astigmatic power vector components C0 and C45, the confidence regions are not fully circular shaped, indicating that the variations in the repeat measurement might be affected by the orientation of the corneal astigmatism. Additional investigation may help to understand the effect of the corneal astigmatism axis on the variation in repeat measurements. Again, all keratometric, corneal back surface and RealPower power vector components show similar variations for the repeat measurements to the Fourier analysis in the 6 mm zone, but systematically lower variations compared to the Fourier analysis in the 3 mm zone. This means that in a clinical environment if we are interested in the ‘robust’ SEQ and astigmatism for keratometry or RealPower derived from Fourier analysis in the 3 mm zone, we should perform a sequence of measurements instead of relying on a single shot, and that we should use any robust metric (e.g., medoids). In contrast, if we are interested in SEQ or astigmatism for keratometry or RealPower or the respective data from Fourier analysis in the 6 mm zone, a single shot might be sufficient as the variability is less.

In Table 3 the explorative data for the mean corneal thickness at the apex and the thinnest point of the cornea are listed together with the corresponding deviations of the repeat measurements from the mean. We see that the Casia shows excellent repeatability for both parameters. Assuming that corneal thickness could be measured clinically with a resolution of 5 microns, fewer than 1% of repeat measurements of CCT and TCT are outside this benchmark. However, from Fig 3 we see that there might be some pachymetry measures with a larger scatter of up to 10 microns from their mean in both directions, and the repeatability of corneal thickness at the thinnest point of the cornea seems to be comparable to the repeatability of corneal thickness at the apex. However, we feel that the variation of repeat pachymetric measures has a minor clinical impact or relevance especially in a context of corneal power evaluation (e.g., to be used for intraocular lens power calculation).

However, the present study has some limitations: A) we used a single centre dataset with repeat measurement from a Casia2 anterior segment tomographer. In a multicentre setup or with different anterior segment tomographers the results might differ. B) as the study was restricted to a sequence of 3 repeat measurements for each eye we were not able to derive the distribution of the variations for the repeat measurements. This information might be helpful for implementing an error propagation model in the future. C) all measurements considered in this study are from a cataractous population scheduled for non-toric intraocular lens implantation (with low to moderate corneal astigmatism). Including repeat tomography measures in a young population and patients with large or excessive corneal astigmatism might give slightly different results. D) In contrast to univariate statistics, there is no unique concept for dealing with multivariate data such as power vectors in cases of non-normality. In this paper we used medoids and confidence regions derived from iterative convex hull stripping, but alternative techniques may produce slightly different results.

**In conclusion**, the present study is one mosaic piece in the potpourri of studies concerning the repeatability of topographers, tomographers and biometers. We consistently used power vectors in terms of spherical equivalent power and astigmatic power vector components C0 and C45 to characterise keratometric power, corneal back surface power, and RealPower representing the cornea as a thick lens. Due to lack of multivariate normality we strictly used nonparametric statistics to represent the variations of the power vector components with repeat measurements. Additional multicentric studies with larger sequences of repeat measurements and enlarged age and astigmatism ranges could help to generalise the results and establish dedicated error propagation models to better understand the impact of measurement uncertainties, e.g., on intraocular lens power calculations with toric lenses.
